# NAD(P)H-Independent Asymmetric C=C Bond Reduction Catalyzed by Ene Reductases by Using Artificial Co-substrates as the Hydrogen Donor

**DOI:** 10.1002/chem.201303897

**Published:** 2013-12-30

**Authors:** Christoph K Winkler, Dorina Clay, Marcello Entner, Markus Plank, Kurt Faber

**Affiliations:** [a] Department of Chemistry, Organic and Bioorganic Chemistry, University of GrazHeinrichstrasse 28, 8010 Graz (Austria) Fax: (+43) 316-380-9840

**Keywords:** alkene reduction, artificial biocatalysis, ene reductases, enzyme catalysis, hydrogen donors

## Abstract

To develop a nicotinamide-independent single flavoenzyme system for the asymmetric bioreduction of C=C bonds, four types of hydrogen donor, encompassing more than 50 candidates, were investigated. Six highly potent, cheap, and commercially available co-substrates were identified that (under the optimized conditions) resulted in conversions and enantioselectivities comparable with, or even superior to, those obtained with traditional two-enzyme nicotinamide adenine dinucleotide phosphate (NAD(P)H)-recycling systems.

## Introduction

**Scheme 1:**
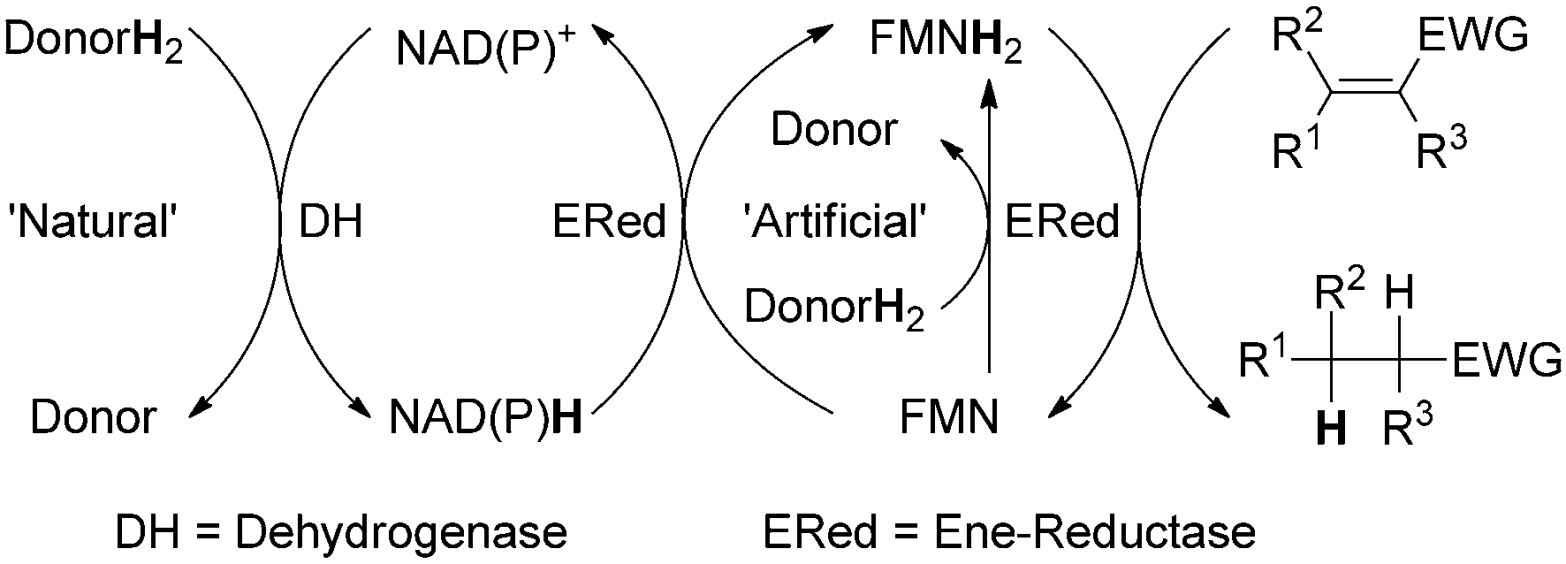
Hydrogen-transfer pathways in the bioreduction of C=C bonds activated by an electron-withdrawing group (EWG): indirect hydrogen transfer from a natural hydrogen donor through nicotinamide catalyzed by a dehydrogenase (coupled-enzyme system); nicotinamide-independent direct hydrogen transfer from an artificial hydrogen donor catalyzed by a single ene reductase (coupled-substrate system).

Flavin-dependent ene reductases from the “old yellow enzyme” (OYE) family have become frequently used for catalyzing the asymmetric reduction of activated C=C bonds.[1, 2] In recent years, these enzymes have been widely applied to the asymmetric synthesis of pharmaceutically relevant targets and industrial intermediates.[3] Despite the excellent stereoselectivities often achieved and the possibility to control the stereochemical outcome of the bioreduction,[4] the overall hydrogen transfer of the commonly employed coupled-enzyme system[5] is rather complex (Scheme 1). After reduction of the substrate, the oxidized flavin cofactor is recycled by NAD(P)H. The latter has to be regenerated through a second redox cycle, requiring an additional dehydrogenase (such as formate, glucose, glucose-6-phosphate, alcohol, or phosphite dehydrogenase), and the corresponding natural co-substrate, which serves as the ultimate hydride source.[5]–[7]

**Scheme 2:**
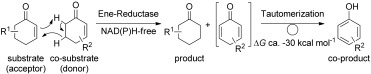
Flavoprotein-catalyzed disproportionation of conjugated enones.

To find more economically advantageous systems, a variety of alternative flavin mononucleotide (FMN)-regeneration systems, such as direct, light-mediated recycling,[8, 9] have recently been developed, which still have to prove their viability in preparative-scale applications.[10, 11] In contrast to the coupled-enzyme method, the coupled-substrate system[5] is appealingly simple because it requires only a single protein together with a suitable, cheap co-substrate, serving as the hydrogen donor for the direct recycling of the flavin cofactor. In this context, we have recently proposed a nicotinamide-independent system, which was developed from the flavoprotein-catalyzed disproportionation of conjugated enones[12]—historically also termed “dismutase activity” or “aromatase activity” (Scheme 2).[13]–[15] The desaturation of the co-substrate is thermodynamically unfavorable because it requires a strong external driving force for the breakage of CH σ bonds, which are not energetically compensated for by the newly formed C=C π bond. However, during the dehydrogenation of cyclohex-2-enones, the newly formed dienone quickly tautomerizes to form the corresponding phenol, which provides a large energy gain of approximately −30 kcal mol^−1^.[13]–[15] Alternatively, elevated temperatures,[16] artificial flavin cofactors with strongly elevated redox potentials,[17] and synthetic nicotinamide analogues have been employed as the hydride source.[18]

In addition to the typical ene reductase activities, OYEs also show NAD(P)H oxidase activity, in the course of which H_2_O_2_ is generated through oxidation of reduced FMNH_2_ by molecular oxygen. Depending upon the type of substrate, hydrogen peroxide thus formed may cause spontaneous Weitz–Scheffer epoxidation of the activated C=C bond,[19, 20] which can be prevented by working under an inert atmosphere.[21]

Although the nicotinamide-independent, coupled-substrate, hydrogen-transfer system could be successfully demonstrated, it suffered from incomplete conversion (≤65 %) due to the enzyme inhibition exerted by the co-product, phenol, which forms a strong charge-transfer complex with FMN.[15, 22]–[27] Although this drawback could be overcome by in situ co-product removal using solid-phase phenol scavengers, the macroscopic polymeric resins caused undesired racemization of chirally sensitive products, such as α-substituted ketones (e.g., **1 a**).[21] To develop a more robust and widely applicable coupled-substrate system, we initiated a search for “artificial” hydrogen donors that would form (quasi)aromatic, but non-inhibiting co-products.

## Results and Discussion

**Scheme 3:**
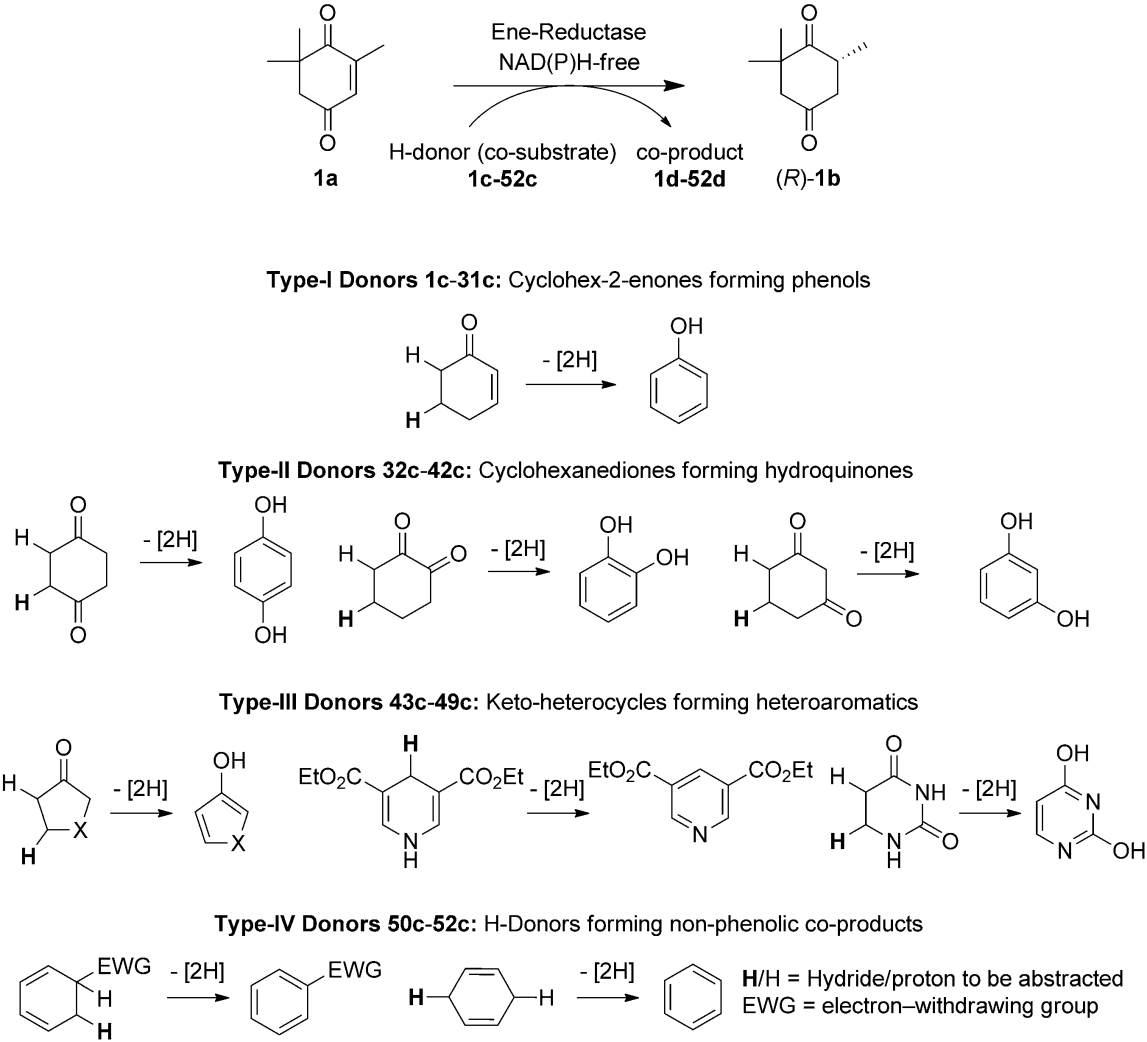
Screening of four different types of hydrogen donor (1 c–52 c) in the NAD(P)H-independent bioreduction of 4-ketoisophorone (1 a).

For our screening of co-substrates, we chose 4-ketoisophorone (**1 a**) as the substrate, which yields, upon bioreduction, chirally sensitive (*R*)-levodione (**1 b**). The latter is an important intermediate for the synthesis of carotenoids (Scheme 3).[28] To account for the broad diversity of ene reductases, OYE1 from *Saccharomyces pastorianus*[29, 30] and XenA from *Pseudomonas putida*[31] were selected as representative candidates due to their distant sequence relationship (27 % identity, 55 % similarity). Both reductases displayed decidedly different activities in preliminary studies.[21] Because activities have been shown to be strongly dependent on the pH of the reaction mixture, hydrogen donors were tested at pH 7.5 and 9. The hydrogen donors can be classified into four groups: type I: derivatives of cyclohex-2-enone, yielding phenols; type II: 1,2-, 1,3-, and 1,4-cyclohexanediones, furnishing hydroquinones; type III: *N*-, *O*-, and *S*-ketoheterocycles, forming heteroaromatics; and type IV: 1,3- and 1,4-cyclohexadiene derivatives, leading to nonphenolic co-products.

**Scheme 4:**
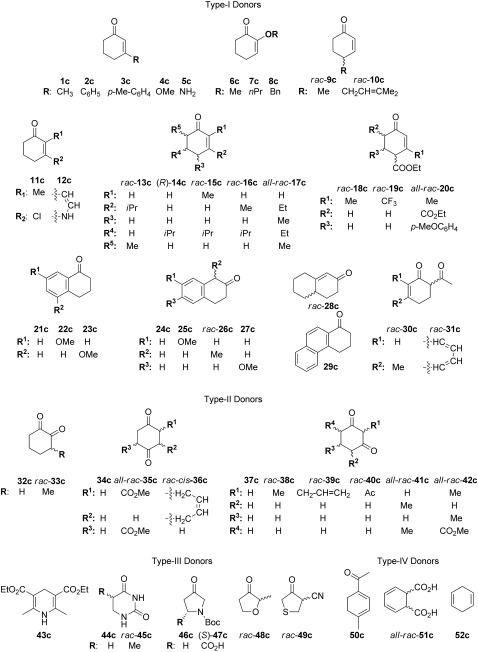
Co-substrates used as hydrogen donors in the NAD(P)H-independent bioreduction of 4-ketoisophorone (1 a) to form (*R*)-levodione (1 b) by using OYE1 and XenA enzymes at pH 7.5 and pH 9 (Boc=*tert*-butoxycarbonyl).

Surprisingly, co-substrates of all types served as hydrogen donors in the test reaction; several even proved to be superior to those previously described (**1 c**, **34 c**).[12] Furthermore, numerous trends could be delineated from the co-substrate screening (Scheme 4):

1) For type I donors, the molecular shape appears to be critical. The small co-substrate **1 c** was a poor hydrogen donor, whereas the larger analogue **13 c** gave conversions of up to 65 %; surprisingly, closely related structures **14 c**–**17 c** were not accepted at all, nor were the 4-substituted derivatives **9 c** and **10 c**. Even more puzzling, compound **3 c** is a weak hydrogen donor (up to 14 % conversion by using XenA at pH 7.5), but **2 c**, lacking a distant *para*-methyl group, shows no activity. Large bicyclic structures **21 c**, **22 c**, and **24 c**–**28 c** (but not **23 c**) acted favorably and proved to be active hydrogen donors. The tricyclic analogue **29 c** was apparently too bulky for this reaction.

2) In addition to steric constraints, electronic activation of the α-carbon atom seems to play a major role, as demonstrated by both co-substrates bearing an additional electron-withdrawing acetyl group in the α-position (**30 c** and **31 c**) being accepted in the reaction. Likewise, compounds **18 c** and **19 c** were found to be weak hydrogen donors. Although **20 c** contains two electron-withdrawing substituents, steric restrictions seem to override the electronic activation. In contrast, enol ethers in the α-position (**4 c**, **7 c**, **8 c**), a β-enamine (**5 c**), or a β-halo derivative (**11 c**) were unsuitable for the reaction, although the α-enol ether analogue (**6 c**) was shown to be a weak donor. Type II derivatives lack a conjugated C=C bond, but possess an enolizable carbonyl group, and hence are generally less suitable for this reaction and none of the tested co-substrates showed conversions of more than 20 %. Interestingly, only 1,2-cyclohexanediones (**32 c**, **33 c**) and 1,4-cyclohexanediones (**34 c**, **35 c**) were accepted by the enzymes, whereas all of the 1,3-cyclohexanediones were inactive, regardless of their substitution pattern or the presence of electron-withdrawing groups (**37 c**–**42 c**).

3) Of the heterocyclic type III co-substrates, none of the six-membered-ring-containing substrates were accepted, including the dihydrouracil derivatives (**44 c**, **45 c**) and the well-known “Hantzsch ester” **43 c**, which has a structural resemblance to reduced nicotinamide and is widely applied as a hydride donor in organocatalytic C=C reduction reactions.[32, 33] In contrast, the majority of the five-membered heterocycles showed moderate to high activities; in particular, *N*-Boc-pyrrolidinone (**46 c**) and 2-methyltetrahydrofuranone (**48 c**) gave 78 and 82 % yields of (*R*)-levodione (**1 b**), respectively. However, low conversion was observed with the thiophenone bearing an additional activating nitrile moiety (**49 c**; 8 % by using XenA at pH 7.5). The surprising performance of five-membered ketoheterocycles as hydrogen donors can be attributed to two things: First, type IV hydrogen donors bear an electron-donating nitrogen or oxygen heteroatom in the γ-position, which facilitates the hydride departure from the β-carbon atom. Secondly, enzyme inhibition occurs due to formation of a charge-transfer complex between FMN and a phenolate anion,[34] which was shown in crystal structures of OYE1 in a complex with *para*-hydroxybenzaldehyde (Protein Data Bank (PDB), entry 1OYB)[35] and of the OYE1 mutant W116A in a complex with 2-methyl-5-(prop-1-en-2-yl)phenol (PDB, entries 4GBU and 4GXM). Clearly, the five-membered hydroxyheteroaromatics formed after hydrogen abstraction from **46 c** and **48 c** result in less favorable π interactions with FMN than phenols or hydroquinones.[24, 34, 36]–[38]

4) Not surprisingly, all co-substrates of type IV, lacking an electron-withdrawing group attached to the alkene moiety (**51 c**, **52 c**), were inactive. Only compound **50 c**, bearing an activating group in the *exo*-position, gave a moderate conversion.

In summary, co-substrates from all four classes were active as hydrogen donors and their reverse (reduction) reaction was observed as a minor side reaction, if a side reaction occurred (<3 % conversion). Steric hindrance plays an important role in the reaction with monocyclic cyclohexenones as the co-substrates, while bicyclic hexenones were more favorable in the reaction. Electronic activation through the presence of an additional electron-withdrawing group (such as an acetyl group) on C_α_ facilitates proton abstraction, whereas electron-donating groups at C_β_ support hydride departure to flavin. In contrast to six-membered heterocycles, five-membered rings were successful co-substrates. The presence of an activating carbonyl group is necessary for the acceptance of a co-substrate.

In the next step, the hydrogen donors that performed best in the co-substrate screening reactions were selected for further optimization studies by using a set of eight ene reductases, which have previously shown the highest acceptance of unnatural co-substrates (other than nicotinamide;[21] Table 1). Generally, all of the selected enzymes were able to accept the six co-substrates (**13 c**, **24 c**, **25 c**, **30 c**, **46 c**, **48 c**) and showed up to 88 % conversion (NCR with **46 c**) in the bioreduction of compound **1 a**. Among the enzymes, XenA exhibited the broadest co-substrate scope, with conversions of 59–78 % with all hydrogen donors except **48 c**. Other favorable enzyme– co-substrate combinations were OYE1 and OYE2 with **25 c** (57 and 59 % conversion at pH 9, respectively) and EBP1 with **48 c** (68 % conversion). Ene reductases from thermophilic microorganisms showed good activities, yielding conversions of up to 64 (CrS with **25 c** at pH 9) and 56 % (*Gk*OYE with **30 c**), although the corresponding stereoselectivities for (*R*)-**1 b** ranged from low to moderate. As previously observed,[21] the α-chiral ketone (*R*)-levodione (**1 b**) is prone to racemization, which occurs even faster at elevated pH and renders substrate **1 a** a challenging candidate.[39] At pH 7.5, however, *ee* values of more than 60 % were generally obtained.

**Table 1:** Selection of the best hydrogen donors and ene reductases in the NAD(P)H-independent reduction of 4-ketoisophorone (1 a) to form (*R*)-levodione (1 b).^[a]^

Co-substrate		pH	OYE1	OYE2	YqjM	XenA	NCR	EBP1	*Gk*OYE	CrS
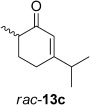	c. [%]	7.5	<1	n.c.	14	34	32	1	15	46
	*ee* (*R*)-**1 b** [%]		n.d.	n.d.	53	73	47	n.d.	91	61
	*ee* (*S*)-**13 c** [%]		n.d.	n.d.	25	40	19	n.d.	18	80
	c. [%]	9	3	3	15	**65**	40	1	47	35
	*ee* (*R*)-**1 b** [%]		n.d.	n.d.	<10	<10	<10	n.d.	6	*rac*
	*ee* (*S*)-**13 c** [%]		n.d.	n.d.	22	85	70	n.d.	96	99
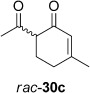	c. [%]	7.5	3	3	44	47	6	2	**56**	45
	*ee* (*R*)-**1 b** [%]		n.d.	n.d.	76	73	86	n.d.	83	77
	c. [%]	9	9	12	14	**64**	20	3	**56**	36
	*ee* (*R*)-**1 b** [%]		19	19	<10	<10	28	n.d.	<10	<10
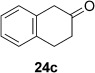	c. [%]	7.5	2	2	2	13	7	7	1	9
	*ee* (*R*)-**1 b** [%]		n.d.	n.d.	n.d.	55	42	66	n.d.	54
	c. [%]	9	41	32	23	**69**	9	5	2	**54**
	*ee* (*R*)-**1 b** [%]		*rac*	*rac*	*Rac*	*rac*	*rac*	n.d.	n.d.	*rac*
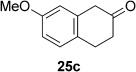	c. [%]	7.5	10	2	4	22	13	16	2	19
	*ee* (*R*)-**1 b** [%]		57	n.d.	n.d.	54	63	57	n.d.	70
	c. [%]	9	**57**	**59**	33	**59**	8	5	1	**64**
	*ee* (*R*)-**1 b** [%]		*rac*	*rac*	*Rac*	*rac*	*rac*	n.d.	n.d.	*rac*
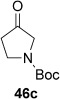	c. [%]	7.5	13	16	30	45	**72**	4	17	31
	*ee* (*R*)-**1 b** [%]		75	68	61	60	70	n.d.	70	65
	c. [%]	9	30	38	35	**78**	**88**	14	49	37
	*ee* (*R*)-**1 b** [%]		<10	<10	<10	<10	12	<10	<10	11
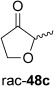	c. [%]	7.5	21	8	6	5	7	24	4	6
	*ee* (*R*)-**1 b** [%]		82	75	74	n.d.	73	78	n.d.	79
	*ee* (*R*)-**48 c** [%]		22	n.d.	n.d.	n.d.	n.d.	51	n.d.	12
	c. [%]	9	35	51	4	12	25	**68**	10	43
	*ee* (*R*)-**1 b** [%]		16	19	n.d.	<10	18	11	16	19
	*ee* (*R*)-**48 c** [%]		99	>99	n.d.	<10	38	92	n.d.	90

[a] Conversions of optimal enzyme–co-substrate combinations are highlighted in bold. Standard conditions: substrate **1 a** (10 mm), enzyme (100 μg mL^−1^), co-substrate **13 c**, **24 c**, **25 c**, **30 c**, **46 c**, **48 c** (10 mm), OYE1 (*Saccharomyces pastorianus*), OYE2 (*Saccharomyces cerevisiae*), YqjM (*Bacillus subtilis*), NCR (nicotinamide-dependent cyclohexenone reductase; *Zymomonas mobilis*), Xenobiotic reductase XenA (*Pseudomonas putida*), EBP1 (estrogen binding protein, *Candida albicans*), GkOYE (*Geobacillus kaustophilus DSM 7263*), CrS (chromate reductase, *Thermus scotoductus* SA-01); c.=conversion; *ee*=enantiomeric excess; n.d.=not determined; n.c.=no conversion.

Co-substrates **13 c**, **30 c**, and **48 c** are chiral and were used in racemic form. With the exception of the chirally unstable β-diketone *rac*-**30 a**, enzymatic dehydrogenation of hydrogen donors *rac-***13 c** and *rac-***48 c** should proceed with kinetic resolution, yielding the corresponding achiral aromatic oxidation co-products and the remaining (slower reacting) co-substrate enantiomer. Indeed, *ee* values of up to greater than 99 % were observed for (*S*)-**13 c** and (*R*)-**48 c**, indicating excellent enantioselectivities with enantiomeric ratios (*E* values) up to >200. Owing to the high enantioselectivities for co-substrates *rac*-**13 c** and *rac*-**48 c**, only 50 % of the hydrogen source is available for the reaction. Consequently, higher conversions should be reached in the presence of two or more equivalents of the co-substrate (Table 2). The apparent imbalance between the *ee* values of (*S*)-**13 c** and (*R*)-**48 c** and the conversion is due to their limited stability after extended reaction times.

**Table 2:** Nicotinamide-independent asymmetric bioreduction of activated alkenes 1 a–6 a by using selected artificial hydrogen donors, 24 c, 25 c, 30 c, 46 c, 48 c (additional data are given in the Supporting Information).

Substrate	Co-substrate [mM]		Enzyme [μg mL^−1^]		pH	Conversion [%]	*ee*_P_ [%]
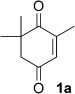	**24 c**	10	XenA	300	9	94	*rac*
	**24 c**	50	CrS	100	9	>99	*rac*
	**25 c**	10	XenA	300	9	>99	<10 (*R*)
	**25 c**	50	CrS	100	9	98	*rac*
	**25 c**	10	CrS	300	9	94	*rac*
	**30 c**	10	*Gk*OYE	300	7.5	93	77 (*R*)
	**30 c**	50	*Gk*OYE	100	9	94	10 (*R*)
	**46 c**	50	NCR	100	7.5	98	88 (*R*)
	**46 c**	50	XenA	100	9	93	27 (*R*)
	**46 c**	50	NCR	100	9	>99	29 (*R*)
	**48 c**	50	EBP1	100	9	>99	21 (*R*)
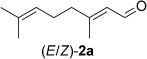	**46 c**	50	NCR	100	9	>99	>99 (*S*)
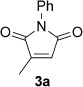	**24 c**	10	XenA	300	9	>99	>99 (*R*)
	**25 c**	50	CrS	100	9	>99	>99 (*R*)
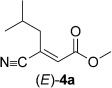	**46 c**	50	NCR	100	9	44	>99 (*S*)
	**48 c**	50	EBP1	100	9	21	>99 (*S*)
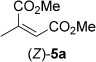	**24 c**	50	CrS	100	9	>99	>99 (*R*)
	**46 c**	50	XenA	100	9	>99	>99 (*R*)
	**48 c**	50	EBP1	100	9	>99	>99 (*R*)
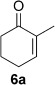	**25 c**	50	CrS	100	9	>99	96 (*R*)
	**30 c**	50	*Gk*OYE	100	9	>99	96 (*R*)

Inspired by these results, the enzyme/co-substrate combinations giving the highest conversions with substrate **1 a** were further optimized with respect to enzyme loading and co-substrate concentration, which finally allowed conversions to reach completion (>99 %) and also improved the enantioselectivities for (*R*)-**1 b** (Table 2). To demonstrate the practical applicability of the optimized system, several types of substrate—enal **2 a**, enone **6 a**, α,β-unsaturated esters **4 a** and **5 a**, and the cyclic imide **3 a**—were tested (Table 2).

(*S*)-Citronellal (**2 b**) was obtained from citral (**2 a**) by using NCR with **46 c** as the hydrogen donor with quantitative conversion and excellent stereoselectivity (>99 % *ee*). Likewise, compound **3 a** was reduced quantitatively by using XenA and CrS at elevated enzyme loading or in the presence of a five-fold excess of **24 c** as the hydrogen donor. With (*E*)-β-cyanoacrylic ester **4 a**, only enzymes NCR and EBP1 were active, and both gave similar conversions and stereoselectivities to the classic NAD(P)H system.[40] Diester **5 a** and α-methylcyclohex-2-enone (**6 a**) were quantitatively reduced with excellent stereoselectivities with various enzyme–co-substrate combinations. The *ee* value of 96 % for **6 b** was caused by imperfect stereoselectivity and not due to racemization, as in case of **1 b**. The absolute configurations of products **1 b**–**6 b** were determined as previously reported,[29, 40, 42, 43] and those of **13 c** and **48 c** were determined through co-injection on a GC with an independently synthesized reference material (see the Experimental Section for details). Aromatic co-products from the biotransformations were identified by co-injection on a GC with commercially available reference compounds **13 d**, **24 d**, **25 d**, and **30 d** and with independently synthesized reference materials **46 d** and **48 d**.

## Conclusion

Four types of H-donor—encompassing more than 50 candidates consisting of cyclohex-2-enones, cyclohexanediones, 5- and 6-membered *N*-, *O*- and *S*-ketoheterocycles and dienes—were screened in the coupled-substrate, nicotinamide-independent bioreduction of C=C bonds by using flavin-dependent ene reductases. Six co-substrates were identified that (under optimized conditions) resulted in conversions and enantioselectivities comparable with, or even superior to, those obtained in the presence of an excess of nicotinamide cofactor or in combination with traditional NAD(P)H recycling.[29, 39, 41]–[44] These results prove the practical applicability of the NAD(P)H-independent, single-enzyme, hydrogen-transfer system by using cheap (commercially available), artificial hydrogen donors. Although the in situ recycling of hydrogen donors is presently not feasible, the co-substrate costs for this reaction are modest.[45]

## Experimental Section

### General

TLC plates were run on silica gel Merck 60 (F_254_). Silica gel 60 from Merck was also used for flash column chromatography. GC-MS analyses were performed on an HP 6890 Series GC system equipped with a 5973 mass selective detector and a 7683 Series injector using a (5 % phenyl) methylpolysiloxane capillary column (HP-5MS, 30 m×0.25 mm, 0.25 μm film). GC-FID analyses were carried out on a Varian 3800 and on an Agilent 7890A by using H_2_ as the carrier gas (14.5 psi). NMR measurements were performed on a Bruker Avance III 300 MHz NMR spectrometer. Chemical shifts are reported relative to trimethylsilane (TMS, *δ*=0.00 ppm) and coupling constants (*J*) are given in Hz.

### General procedure for the nicotinamide-independent anaerobic enzymatic C=C reduction reaction

An aliquot of the isolated enzyme (OYE1, OYE2, CrS, EBP1, NCR, XenA, YqjM, *Gk*OYE; protein purity >90 %, protein content in reaction 100 μg mL^−1^) was added to a screw-top glass vial (2 mL) containing a degassed buffer solution (0.8 mL, 50 mm, tris(hydroxymethyl)aminomethane**⋅**HCl (TrisHCl) buffer; pH 7.5 or pH 9), the substrate (**1 a**–**6 a**, 10 mm), and the hydrogen donor (**1 c**–**52 c**; 10 mm). The vial was flushed with argon, and sealed with a teflon-coated septum and a lid. The mixture was shaken for 24 h at 30 °C and 120 rpm by using an Infors Unitron shaker and the products were extracted with ethyl acetate (2×0.7 mL). The combined organic phase was dried over Na_2_SO_4_ and analyzed on a GC to determine the conversion and stereoselectivity. On a preparative scale, products could be easily separated from excess hydrogen donor and phenolic byproducts by simple silica gel filtration due to the large difference in *R*_f_ values.

### Synthesis of α-(+)-3,4-epoxycarene[46]

A solution of *meta*-chloroperbenzoic acid (1.037 g, 6.0 mmol in CHCl_3_ (12 mL)) was added dropwise to a stirred solution of (+)-carene (0.508 g, 3.7 mmol) in chloroform (6 mL) over a period of 75 min. The reaction was stirred for a further 40 min and then quenched with aqueous sodium bisulfite (40 %, 2 mL). The organic layer was separated, washed with saturated aqueous NaHCO_3_ (15 mL) and brine (15 mL), dried with Na_2_SO_4_, and concentrated by evaporation of the solvent to give α-(+)-3,4-epoxycarene as a light yellow oil (0.561 g, 3.68 mmol).

### Synthesis of (*S*)-3-isopropyl-6-methylcyclohex-2-enone [(*S*)-13 c][47]

Crude α-(+)-3,4-epoxycarene (355 mg, 2.6 mmol) was dissolved in dichloromethane (10 mL) and cooled to −78 °C (N_2(l)_/EtOH). Trimethylsilyl triflate (TMSOTf; 44 μL) was added and the reaction was stirred for 3 h. Saturated aqueous NaHCO_3_ (5 mL) and diethyl ether (10 mL) were then added. The organic layer was separated, washed twice with brine (10 mL), dried with Na_2_SO_4_ and concentrated by evaporation of the solvent to give (*S*)-3-isopropyl-6-methylcyclohex-2-enone [(*S*)-**13 c**; 45 mg, 0.3 mmol, 12 %, 25 % *ee*]. Spectroscopic data were in agreement with those of the commercially available reference compound *rac*-**13 c**.

### Synthesis of methyl 5-methyl-4-oxotetrahydrofuran-3-carboxylate[48]

Methyl L-lactate (1.0 g, 9.8 mmol) was dissolved in diethyl ether (4 mL) and added to a cooled (−38 °C, N_2(l)_/EtOH) suspension of NaH (267 mg, 50 %, 5.6 mmol) in diethyl ether (6 mL). The mixture was allowed to warm to 0 °C and stirred for 20 min at this temperature. The solvent was evaporated and a solution of methyl acrylate (1 mL, 11.0 mmol) in DMSO (4 mL) was added to the residue. The reaction was stirred for 20 h at ambient temperature. The mixture was poured into cold, aqueous sulfuric acid (5 %) and extracted three times with diethyl ether (40 mL). The organic layers were combined, washed with saturated aqueous NaHCO_3_ (20 mL) and brine (20 mL), dried over MgCO_3_ and concentrated by evaporation of the solvent. The residue was purified by column chromatography (hexane/ethyl acetate 20:1), which yielded methyl 5-methyl-4-oxotetrahydrofuran-3-carboxylate (990 mg, 6.26 mmol, 64 %). TLC results were viewed by using a KMnO_4_ staining solution or UV_254_ (*R*_f_=0.34, hexane/ethyl acetate 2:1).

### Synthesis of (*R*)-2-methyldihydrofuran-3(2*H*)-one [(*R*)-48 c][48]

Methyl 5-methyl-4-oxotetrahydrofuran-3-carboxylate (200 mg, 1.3 mmol) was added to sulfuric acid (10 %, 5 mL) and the mixture was stirred for 3.5 h at 70 °C. The reaction mixture was then cooled to ambient temperature, poured into saturated aqueous NaHCO_3_ (50 mL), and extracted three times with ethyl acetate (30 mL). The organic layers were combined, washed with saturated aqueous NaHCO_3_ (20 mL) and brine (20 mL), dried with MgSO_4_, concentrated by evaporation of the solvent and purified by column chromatography (hexane/ethyl acetate, 5:1) to yield (*R*)-2-methyldihydrofuran-3(2*H*)-one [(*R*)-**48 c**, 20 mg, 0.2 mmol, 94 % *ee*]. TLC results were viewed by using a KMnO_4_ staining solution (*R*_f_=0.36, hexane/ethyl acetate 2:1). Spectroscopic data were in agreement with those of the commercially available reference compound *rac*-**48 c**.

### Preparation of *tert*-butyl 3-oxo-2,3-dihydro-1*H*-pyrrole-1-carboxylate (46 d)

An aliquot of isolated NCR (protein purity >90 %, protein content in reaction 200 μg mL^−1^) was added to 30 screw-top glass vials (2 mL) containing a degassed buffer solution (0.8 mL, 50 mm, TrisHCl buffer; pH 7.5 or pH 9), 4-ketoisophorone(**1 a**, 30 mm), acting as the hydrogen acceptor, and *tert*-butyl 3-oxopyrrolidine-1-carboxylate (**46 c**; 10 mm). The vials were flushed with argon, sealed with a teflon-coated septum and a lid. The mixtures were shaken for 24 h at 30 °C and 120 rpm by using an Infors Unitron shaker. After the transformation, all phases were collected and the products were extracted with ethyl acetate (2×30 mL). The combined organic phase was dried over Na_2_SO_4_, concentrated, and the product was purified by column chromatography (hexane/ethyl acetate, 5:1) to yield *tert*-butyl 3-oxo-2,3-dihydro-1*H*-pyrrole-1-carboxylate (**46 d**; 10.5 mg). TLC results were viewed by using a KMnO_4_ staining solution (*R*_f_=0.65, hexane/ethyl acetate 2:1). ^1^H NMR (300 MHz, CDCl_3_): *δ*=8.33 (d, *J*=4.1 Hz, 2 H), 5.65 (d, *J*=4.2 Hz, 2 H), 4.01 (s, 2 H), 1.54 ppm (s, 9 H).

### Synthesis of 2-methylfuran-3(2*H*)-one (48 d)

2-Methyldihydrofuran-3(2*H*)-one (**48 c**, 2 g, 20 mmol, 1.93 mL) was dissolved in dry THF (100 mL) and cooled to −80 °C (liquid N_2_/EtOH) under an argon atmosphere. *N*,*N*-Diisopropylethylamine (7.78 g, 60 mmol, 10.4 mL) was then added over 10 min and the mixture was stirred for 10 min, followed by slow addition of trimethylsilyl trifluoromethanesulfonate (8.89 g, 40 mmol, 7.23 mL) over a further 10 min. The mixture was stirred and kept at between −60 °C and −80 °C for 90 min and then allowed to warm to room temperature over 90 min. The solution was then cooled to −60 °C, and *N*-bromosuccinimide (4 g in 50 mL of dry THF) was added, turning the yellow solution red. The mixture was stirred for 60 min at this temperature and then the reaction was quenched by addition of water (100 mL) and dichloromethane (100 mL). The phases were separated and the aqueous phase was washed with dichloromethane (3×50 mL). The combined organic phases were dried over Na_2_SO_4_, concentrated, and the resulting oil was immediately purified by column chromatography (hexane/ethyl acetate, 10:1) to remove any residual base. This yielded 4-bromo-2-methyldihydrofuran-3(2*H*)-one (1.35 g), which is unstable in concentrated form and thus was immediately used for the next step.

4-Bromo-2-methyldihydrofuran-3(2*H*)-one (330 mg, 1.9 mmol) was dissolved in ethyl acetate (15 mL). LiBr (646 mg, 7.5 mmol) and Li_2_CO_3_ (562 mg, 7.5 mmol) were then added and the mixture was added to a G30 Anton Paar microwave reaction vessel. The reaction was heated for 5 min at 180 °C by using an Anton Paar Monowave 300 machine. The pH of the mixture was brought to 7 by using aqueous HCl (1 %) and the phases were separated. The organic phase was dried over Na_2_SO_4_, concentrated, and purified by repeated column chromatography (hexane/ethyl acetate 8:1 and *n*-pentane/diethyl ether 4:1) to yield 2-methyldihydrofuran-3(2*H*)-one (**48 d**, 15 mg). TLC: *R*_f_=0.32, hexane/ethyl acetate 6:1; ^1^H NMR (300 MHz, CDCl_3_): *δ*=8.21 (d, *J*=2.4 Hz, 1 H), 5.68 (d, *J*=2.5 Hz, 1 H), 4.45 (q, *J*=7.2 Hz, 1 H), 1.48 ppm (d, *J*=7.2 Hz, 3 H).
